# Filio-parental violence analyzed through the Spanish press (2010–2020). Child-to-parent violence: A case of family violence

**DOI:** 10.3389/fsoc.2023.985173

**Published:** 2023-03-08

**Authors:** Juan Martínez Torvisco, Monika Wichrowska, Monica Pellerone

**Affiliations:** ^1^Department of Psicología Cognitiva, Socialy Organizacional, Universidad de La Laguna, Santa Cruz de Tenerife, Spain; ^2^University of Warsaw, Warszawa, Poland; ^3^Faculty of Human and Social Sciences, Kore University of Enna, Via Cittadella Universitaria, Enna, Italy

**Keywords:** violence, child-to-parent violence, newspapers, family relationships, parents, qualitative analysis

## Abstract

Violence exercised by children against their parents has increased significantly, affecting society both directly and indirectly. This type of violence is one of the least investigated and least exposed by the media. The aim of this work is to examine how the media have portrayed this problem, by means of a qualitative methodology and a multidimensional analysis of journalistic and literary texts. News related to violence committed by children against parents published from 2010 to 2020 were sourced from the following Spanish newspapers: El País, ABC, El Día, El Mundo, La Vanguardia, El Periódico, La Provincia, Canarias 7, 20 Minutos, and Diario de Avisos. The text corpus was subsequently analyzed with the open access software IRaMuTeQ. The results show a total of 204232 words (number of occurrences), of which 4561 appear only once (number of hapaxes). The words “violence,” “father,” and “son” are those that appear most frequently in the analysis of the texts and present a strong co-occurrence among them. It should be noted that child-to-parent violence is described as a problem confined to the family environment. The importance of prevention strategies against violence within the family context is shown in this paper.

## 1. Introduction

Family violence is a very important problem in society. It has various facets and can be carried out in various ways. Among them, the best-known types are child violence and gender violence, in particular violence against women (Souza-Leal et al., 2018), which has been studied for many years and against which there has been major opposition. In the last decade, aggressions perpetrated by young people against their parents or caregivers have also become a source of social alarm.

In recent years, more recognition has been given to this type of violence, which is known as filio-parental violence (hereinafter FPV). Although there have been some research teams studying the subject (Calvete et al., 2011; Ibabe and Jaureguizar, 2011; Cano-Lozano et al., 2021), and 2013 saw the establishment of the Spanish Society for the Study of Filo-Parental Violence (SEVIFIP), this type of family violence was the least studied in the last decade and seemed to have less weight in media coverage.

Today, because of an increase in cases of FPV, more research is being carried out to understand its possible causes and what the most effective interventions are, above all for its prevention and treatment. The limited research there has been on this topic is probably due to some methodological issues in the identification of the aggression itself: some research demonstrates both verbal violence that concludes with threats and attacks that lead to physical injuries; likewise, other research considers the subjective perception only of verbal threats or abuse (Franchini et al., 2020). Furthermore, there are difficulties in identifying instruments for detecting the violence suffered.

However, evidence has been found of screening instruments with psychometric properties suitable for use in correlational and quasi-experimental studies. For a systematic review, see the research by Ibabe (2020) as well as the application on Spanish adolescents of the Child-to-Parents Violence Questionnaire (CPV-Q) by Contreras et al. (2019) and its adaptation to Chilean youth by Bueno et al. (2020). Furthermore, there is a meta-analytic review carried out by Gallego et al. (2019).

The reasons why the term FPV is becoming more widely known are diverse. Jiménez (2017) proposes that there is “a greater social awareness regarding family violence, due to the increase in the number of complaints, due to their mediation, due to legislative advances or due to new institutional figures and specialized services” (p. 16). Although there is no such legislation for this type of violence in Spain, there is a regulation (1/2,010) issued by the State Attorney General's Office (Fiscalía General del Estado) on the treatment of minors who mistreat their progenitors.

However, attention should be paid to how the concept of FPV has evolved over time, since it has not always been treated in the same way. Early definitions used at the end of the 20th century were very vague and not so descriptive. In the United States, at the end of the 1970's, the term “battered parent syndrome” appeared in reference to those parents who suffered physical attacks and threats, both verbal and non-verbal (Harbin and Madden, 1979). In Spain, Urra (1994) groups the behaviors young aggressors present in terms of tyrannical attitudes (the purpose of which is to cause permanent damage through threats and aggressions), use (especially in economic terms), and detachment (to convey that they are not loved). Cottrell (2001) defines FPV as any harmful act to gain power and control over parents, adding that it can be physical (hitting, punching, pushing, throwing objects, etc.), financial (theft of belongings or money, getting into debt, destroying property, etc.), or psychological (privacy, threatening, blackmailing, etc.). Therefore, FPV is exercised by the child intentionally with the desire to cause damage, harm, and/or suffering to their parents, repeatedly, over time, and with the immediate end of obtaining power, control, and dominion over their victims to get what they want through psychological, economic, and/or physical violence (Aroca, 2020).

In general, youth-to-parent aggression (YPA) can be defined as abusive behavior perpetrated against a parent by a legally recognized child who usually lives in the family home (Holt, 2016; Ibabe, 2020).

Moreover, it is necessary to add the final definition of FPV proposed by the SEVIFIP Group of Experts, cited by Pereira et al. (2017, p. 220): “repeated behavior of physical, psychological (verbal or nonverbal) or economic violence, directed toward the parents or the people who occupy their place. Excluded are one-off aggressions that occur in a state of diminished consciousness which disappear when upon recovery (intoxications, withdrawal syndromes, delirious states or hallucinations), those caused by (transient or stable) psychological disorders (autism and severe mental deficiency) and parricide without history of previous aggressions.”

These definitions display different characteristics, such as the intention to cause harm, the presence of a legally recognized child, or a cohabitating parent in the same house. Unfortunately, in the last decade, the use of different conceptual and operational definitions to study YPA has affected the ability to identify risk factors for this type of abuse (Simmons et al., 2018).

In addition to the conceptual aspects, it is important to address the research implications of this complex phenomenon arising from the difficulty in obtaining objective data of subjective facts. This probably explains the limited research on this topic, i.e., the identification of the source of aggression; most studies have obtained data from a single source of information (parents or children).

The topic of “violence” is diverse and can be analyzed in various ways, but the aggression of young people toward their parents or caregivers has become a cause of social alarm in the last decade.

To this end, the bibliographic review by Calvete and Perreira (2019) is interesting, as it brings together the main research conducted from 2010 to 2018, underlining how most of the studies—in the Spanish context—have obtained data from a single source of information (i.e., parents or children). The authors underline the enormous variability of the phenomenon because of gender and age variables and, above all, the source of information, since in some cases the informants are the children themselves and in others the parents. Del Hoyo et al. (2020) highlight the complexity of the variables involved in the development of CPV.

As for age, there are variations depending on “the methodology employed, the sample inclusion parameters and the criteria used to determine the age of the children” (Aroca et al., 2014, p. 163). Research conducted through the analysis of reports of violence indicates that the peak age of children who commit FPV is between 14 and 17 years, decreasing in frequency at higher ages (Simmons et al., 2018). Research on the phenomenon distinguishes between the adolescent and young adult phases, which are characterized by distinctive elements in the manifestation of aggressive or violent behavior, such as impulsivity (Ibabe et al., 2007; Calvete et al., 2011), difficulty controlling anger (Cuervo and Rechea, 2010; González-Álvarez et al., 2011), and low frustration tolerance (Cuervo et al., 2008; Cuervo and Rechea, 2010); this is evident in the characteristics of the victims and in the predictive psychosocial factors (Suárez-Relinque and del Moral Arroyo, 2020).

In this study, we will demonstrate this topic's academic background and a list of a few studies and also engage with the various statistics gathered on FPV. For example, the literature emphasizes that during adolescence, sons tend to manifest physical and, above all, psychological violence. Notably, the study of Boxer et al. (2009), which examines 232 mother–adolescent dyads in detail—with sons and daughters ranging from 11 to 18 years old—employs data that is drawn from a database of families referred for clinical treatment of emotional and behavioral problems in their adolescent children. The results of this research, which examines only the parents' perspective, demonstrate that 35.2% of mothers declared violence committed by their sons and 29.1% by daughters, and 28.7% of fathers reported violence committed by their sons and 15.5% by daughters.

In contrast, the study of Calvete et al. (2011) reveals that in a group of 1,427 teenagers (728 girls and 682 boys; 12–17 years), 7.2% manifested physical violence and 65.8% psychological violence toward their parents. The results show that while verbal forms of aggression are relatively frequent, physical attacks are committed by few minors. However, although these percentages are small, they are significant given the nature of the behaviors included. Notably, the data are similar to those obtained in samples from other countries. Similarly, Calvete et al. (2013) show that in a group of 2.672 teenagers (of which 52% were girls) between 12 and 18 years of age, 10.7% committed physical violence and, above all, 92.7% committed psychological violence toward their parents. Furthermore, Ibabe (2014) evaluation of 485 adolescents (of which 55% were boys; 12–18 years) showed that adolescents committed emotional (46%) and psychological (31%) violence, and to a lesser extent psychic (21%) and economic violence (19 %). It was also found in this study that adolescent physical violence decreased with age; that is, many adolescents who begin to show antisocial behavior during adolescence do not continue to do so in adulthood. However, in contrast, it has also been found that other adolescents exhibit antisocial behavior early on, which persists throughout their life (Moffitt, 1993); this study confirms that behavioral problems increase with age (at least during adolescence), and these results are consistent with those of previous studies.

More recently, Calvete and Veytia (2018) study on 1.417 teenagers (57% girls; 14–19 years) reports a rate of 6.4% of physical violence toward mothers and 6.1% toward fathers, and 87.2% of psychological violence toward mothers and 72% toward fathers. Data analyses have supported the existence of two types of violence: psychological aggression and physical aggression, both toward the mother and/or the father. On the other hand, few studies have revealed the presence of violent behavior among young adults toward their parents; for example, Lyons et al. (2015) study of a group of 365 university students (18-24 years) shows the occurrence of psychological violence (75,8%) and physical violence toward mothers (6,3%) and fathers (5.5%).

In relation to the gender variable, Ibabe et al. (2020) conducted a study on a group of 847 college students, ranging from 18 to 25 years of age, which when using technical abuse criteria shows that daughters were more frequent perpetrators of psychological child-to-parent violence (CPV) than sons. This result is consistent with the findings of another study by Rico et al. (2017), which examines a sample composed of 934 students aged between 13 and 21 years. This study confirms the previous literature (Calvete et al., 2011) that demonstrates how adolescent boys tend to physical violence while adolescent girls perform more acts of verbal or psychological violence.

Furthermore, the recent study by Cano-Lozano et al. (2021) has measured CPV in a group of 2245 young people (52.8% girls) aged between 18 and 25 years, showing that more than half of the youths reported having exercised violent behavior toward their parents within at least a month (65.2% toward the mother and 59.4% toward the father). The type of violence manifested is mainly psychological, characterized by behavior of control (36.5%) and domination (43%), followed by economic violence (which ranged from 12 to 16.6%). More girls than boys exercised psychological violence toward fathers and mothers and controlling/dominating behaviors toward mothers. Conversely, more boys than girls exercised physical and economic violence against parents. The study confirms interesting results on gender differences—specifically, the self-assessments reveal differences in psychological aggression against the mother, which occurred at a higher percentage in the group of girls than in the group of boys.

Therefore, the Spanish literature emphasizes the absence of significant gender differences in CPV perpetration, except in psychological aggression, which is more characteristic of girls than boys (Ulman and Straus, 2003; Calvete et al., 2011, 2013; Ibabe, 2015). However, when the violence is severe, boys appear to be the most frequent perpetrators (Moulds et al., 2019), while with respect to mild physical violence, differences between boys and girls are not found or are very small (Ibabe and Jaureguizar, 2011).

As for the data emerging from interviews with parents, the results underline the presence of greater physical aggression perpetrated by boys compared to girls. Moreover, consistently with the data emerging from the interviews with the children, adolescent boys tend to use more physical violence while adolescent girls commit more acts of verbal violence, and mothers experience more verbal violence than fathers, and this is more so from daughters than from sons (Walsh and Krienert, 2007; Aroca et al., 2014).

Another Spanish study reveals that other than the prevalence of physical aggression against mothers, which was higher among girls, there were no significant differences in physical aggression against parents (Calvete et al., 2013). This may be due to the fact that physical assaults are perceived differently, i.e., in a more pronounced way when exercised by boys, because they generally have greater physical strength, making their attacks more threatening and harmful.

These results lead to the hypothesis that gender differences related to CPV change according to the age of the offender, suggesting greater differences in the transition from adolescence to young adulthood, and according to the sources consulted (mother, father, and/or or children).

Certainly in this latter respect, the bibliographic review by Calvete and Perreira (2019) highlights the presence of a single study among those collected from the period between 2010 and 2018, whose source of information is both the parents and children (Calvete et al., 2017). The main objective of this study was to examine the consistency between the parent reports and child reports when reporting on child-to-parent violence in a community sample. There is less data obtained from parents than from the children, except in the cases of psychological and severe physical aggression against the father. These results suggest that parents may underestimate the violence they suffer, as has been suggested in the few previous studies that evaluated discrepancies between these reports (Pagani et al., 2004, 2009). Parents may downplay some of their sons” and daughters' behaviors, as occurs in other forms of abuse, such as gender-based violence. Additionally, some parents may feel uncomfortable admitting that their sons and daughters treat them unfairly.

### 1.1. Victims and family context

FPV is usually a problem that does not spread outside the family environment, which is believed to be due to shame, rejection by society, or even fear of the reaction that the children may have. As a result, in order to protect the family environment, the victims prefer to keep quiet and to not transmit domestic occurrences outside the home (Martínez et al., 2015). Within this environment, many women who have decided to be single mothers—those who are separated or divorced and are therefore more likely to raise their children alone—thus assume responsibility for the upbringing and education of their children. They often have feelings of guilt when they cannot control their children's attitudes and believe that their children's behavior is determined by their relationship with them (Pérez and Pereira, 2006). These abusive attitudes on the part of the children include insults, threats, harm, physical and psychological violence, and attempts at humiliation, which can cause devastating short- and long-term damage to the human being. These damages include emotional discomfort, physical and mental health problems, work difficulties, and problems in social and family relationships (Holt, 2016).

Currently, when children make intense displays of negative behavior such as shouting, insults, and scandals, parents tend to maintain a conciliatory attitude to reduce stress at home. Parents become frustrated and more rigid, and these expressions in the family environment cause irritability in the children. The children, knowing the extent of their power, increase both the frequency and intensity of their violent behavior, making their parents feel powerless. These hostile interactions can continue to escalate: they begin with insults, evolving into threats and breaking objects, and over time they become increasingly serious physical attacks (Pereira and Bertino, 2009). The more extreme the children's behaviors, the greater the tendency for parents to give in to maintain calm. This is the so-called circle of children-to-parents violence. In most cases, these aggressive behaviors only occur in the family context (Aroca et al., 2014). As for the variables that may be related to the aggressor themself, the most studied have been gender and age. As aforementioned, studies on violence against fathers and mothers have not found significant differences between genders (Ulman and Straus, 2003; Ibabe and Jaureguizar, 2011).

Although there has been some variability in the findings up to this point, gender is a variable in which there is increasingly less difference being detected, mainly due to egalitarian education. The vast majority of research indicates that boys and girls exert violence against their fathers and mothers to the same extent. However, there are differences in terms of the type of violence exercised, with sons exercising the most physical violence (Walsh and Krienert, 2007) and daughters exercising the most psychological violence (Aroca et al., 2014). Similar results were obtained in a recent study conducted on FPV during the COVID-19 pandemic (Abadías-Selma, 2020). This difference is probably due to the fact that boys show higher levels of impulsivity than girls: girls, in the first phase, reach the highest levels of sensation seeking, and thereafter the search for sensation diminishes rapidly; in contrast, boys manifest an increased impulse control more gradually than girls (Shulman et al., 2015; Ballarotto et al., 2017).

Victims of FPV are usually mothers (although it is the fathers who receive the highest degree of hostility) who, as a consequence of the aggressions, suffer stress and tension, as well as feelings of frustration, helplessness, and fear (Sempere et al., 2006; Aroca et al., 2014; Wilcox and Pooley, 2015b). Both mothers and fathers are reluctant when it comes to reporting the situation for various reasons, including having been threatened by the aggressors, fear of the consequences that the report would bring, or wanting to maintain their image in the social environment (Jiménez, 2017). In addition, and especially in the case of mothers, due to double victimization and stigmatization, it is considered in the societal context that they have failed due to not having adequate parenting skills (Wilcox and Pooley, 2015a). In fact, the literature emphasizes that experiences with caregivers during childhood and the resulting attachment styles are linked to capacities for emotional regulation (Heshmati and Pellerone, 2019).

The role of the media in the dissemination and prevention of this type of violence has not been very significant. Violence in the family environment, as well as gender violence, have a long history of research that analyzes their relationship with the media; however, this is not the case with FPV. The media exert great influence over people, and although this is not enough to produce changes in beliefs or behavior, from one day to another, the effect caused by the messages emitted repeatedly and continuously over time must be considered (Rolle et al., 2014). In fact, social media use can be considered a “way of being,” especially for young people (Griffiths and Kuss, 2017).

Moreover, as Wilcox and Pooley (2015a) point out, the self-guilt experienced by some parents who are victims of FPV can be amplified by the messages transmitted by the media. Indeed, media outlets report on topics that they deem appropriate (either due to the novelty, severity, or evolution of events) in accordance with their ideologies and orientations, and in cases of violence, they do so in a superfluous way. This allows the recipient to be informed of the most basic aspects, but it simultaneously shields them from the full reality of the problem, so as not to cause them anguish (Valdemarca and Bonavitta, 2011).

Although it is true that FPV does not have as much resonance in Spain as gender violence may have, it has been openly addressed on TV shows such as “Hermano Mayor,” which has been broadcast since 2009 with a share of 2 million viewers and with an average audience share of 11.2% during its broadcast with Pedro García Aguado and Sonia Cervantes. The protagonists of the program are young people aged between 18 and 25 years old—who have problems with violent behavior and, sometimes, drug use—and their relatives. This program intends to provide the viewer with a frame of reference to help them in the education of their children.

The study of language in the written press is not new—it is sometimes called lexicometry. Tiscareño-García and Miranda-Villanueva (2020) have already used qualitative content analysis and framing as a conceptual framework, establishing categories for the language of violence against women. Moreover, during our exploration of reports in the Spanish written press, we observed that despite the varying amounts of news about FPV published among the different newspapers, the number generally increased over the years. However, FPV appears to remain a taboo subject in Spanish society, with little research and visibility. The media can provide a means of attracting attention to this problem and expanding the transmission of information to society (Bullock, 2007).

### 1.2. Objectives

The objectives of this study were to reflect the vision of FPV given in written press reports, which were collected through lexicometric analysis; to learn, through the aforementioned analysis, the words that were chosen to describe FPV in the Spanish written press in the years from 2010 to 2020; to analyze the news related to this violence in different Spanish newspapers, including “ABC,” 'La Vanguardia,” “El País,” “El Mundo,” and “20 Minutos” (mainland) and “La Provincia,” “El Día,” “Canarias7,” and “Diario de Avisos” (islands); and to discover if there were differences between newspapers in the treatment of FPV and to discern the relationship between the terms “father” and “violence” based on the studied variables together with the texts. Furthermore, we were interested in understanding the “clusters,” lexical classes, or categories that were formed when studying the full text corpus.

## 2. Materials and methodology

In order to explain the results, we almost always appeal to what happened in the past, which, although a good predictor, may not coincide with our results. The introduction given to previous research (Ibabe et al., 2007, 2020; Ibabe, 2020; Tiscareño-García and Miranda-Villanueva, 2020) and its results is meant to highlight the wider interest in our topic of research. The literature emphasizes how current the FPV phenomenon is, and therefore an analysis that differs from the purely descriptive one obtained through questionnaires or open interviews makes sense. The present paper is presented through the perspective of journalistic discourse analysis. For the analysis of discourse from lexical parameters, we used the IRaMuTeQ software, which, through a system of coding and statistical multidimensional analysis, allowed us to deepen and categorize the lexical worlds present in the media discourse. The use of software is not a method of data analysis in itself, but a tool to process the data; therefore, its interpretation is a crucial responsibility of the researchers.

The data were interpreted through lexicometric statistical analysis with terms from the same subject. The aim was to highlight the “usual lexical worlds”. A lexical world is the statistical trace of a place in the vocabulary, which is usually considered important to a writer. Lexical worlds (not probabilistic, i.e., for convenience) can then be studied through the analysis of the organization and distribution of co-occurring main words in the simple sentences of a text. This methodology focuses on the statistical distribution of successions of words that make up the sentences of a text; this does not consider the syntax of the discourse, but only the “co-occurrence” or simultaneous presence of several main words (nouns, adjectives, verbs) in the same sentence, eliminating relational words from the analysis—namely, conjunctions, prepositions, and articles (Reinert, 1983). A clear three-step model for text analysis has been provided: data collection, data modeling, and data analysis.

### 2.1. Text corpus

The preparation and tabulation of the text was conditioned by the presence or absence of the concept of FPV; therefore, only those texts in which the concept explicitly appeared were selected. To prepare the text, we used the procedure of selecting main words, verbs, and nouns, and the analysis software lemmatized the words to ensure there was no overlap; however, we applied the root for the final count.

The selection of the terms for analysis was carried out jointly by the authors, together with the research team with whom we worked during the textual statistical analysis. The terms were chosen by a majority of the members; if a word created uncertainty or had a double meaning, it was eliminated from the text. The perspective guiding the choice was a clear relationship with the phenomenon of domestic violence. Although the program detected words that were irrelevant to this type of violence, as the authors of the texts were responsible for selecting the texts, they were kept as referents of the newspapers where they were published regardless of their ideological tendency.

The database under analysis included news related to FPV that were published between 2010 and 2020 by the Spanish written press. In total, there were 119 news items obtained from 9 Spanish newspapers: El País, ABC, El Día, Diario de Avisos, La Vanguardia, El Mundo, Canarias7, La Provincia, and 20 Minutos.

### 2.2. Instruments

A statistical analysis of textual data (*analyse statistique des données textuelles*) was used, which is a lexicometric approach that originated in French-speaking countries and is still strongly rooted in continental Europe. It is mainly based on the comparison of lexical profiles and thus on the distribution of word occurrences without going through the direct reading of the text (for this reason, it is also defined as “automatic”).

To carry out the statistical data analysis, the Interface de R pour les Analyses Multidimensionnelles de Textes et de Questionnaires (IRaMuTeQ) program was used, which allows the analysis of texts and questionnaires (Ratinaud, 2009). We used version 0.7 Alpha 2 and version 3.2.1 of R (free software), which executes the instructions directly without a prior compilation of the program into machine language instructions. This software was created by the Laboratoire d'Études et de Recherches Appliquées en Sciences Sociales (LERASS) of the University of Toulouse, based on the classification method of Reinert (1983). It performs content analysis, lexicometry, and discourse analysis using the R software interface, which was created by Ihaka and Gentleman (1996) to provide an environment for statistical analysis in scientific research.

The IRaMuTeQ software allows the multidimensional analysis of texts of different natures, such as official texts, web pages, news, laws, and open-response questions of questionnaires, identifying and establishing the connections that exist between them (Zuur et al., 2009).

### 2.3. Procedures and analysis of information

Given that this was a lexicometric analysis of texts, the first step consisted of an exhaustive search of all the articles on FPV published in selected Spanish newspapers during the focus years of the study (2010–2020) for analysis by the IRaMuTeQ program. For this, the archives of each newspaper were accessed and, by use of word search filters (violence, child-to-parent violence, newspapers, family relationships) that were limited to the years of publication and the keywords associated with the subject in question, the articles were selected one by one after verifying that they were relevant to the object of study. The FPV manual was incorporated complete with the same coding as the newspapers. Subsequently, so that the categorical variable would be the year of publication, the texts were grouped by year using an 8-bit Unicode Transformation Format (UTF8) to serve as the input for the IRaMuTeQ program as a text corpus, i.e., the chosen set of news items formed the corpus of the text on which the analyses were performed.

The program carries out a stemming process, by which it reduces words to their roots, classifying them syntactically. This process facilitates the analysis and interpretation of the results, since it allows to choose what type of words to include in each analysis. Therefore, in our procedure, several lexicometric analyses were carried out in which both nouns and verbs were included: word frequencies, word cloud, analysis of specificities, descending hierarchical classification (Reinert Method), and analysis of similarities.

## 3. Results

The results obtained are divided into four sections, which correspond to the lexicometric analyses carried out: frequency and word cloud, specificity analysis, descending hierarchical classification, and similarity analysis. In the first section, we explore the characteristics of the text and the most frequent words. In the second section, we examine the relationship between the most frequent words and the year variable. The third describes the classifications of words found in the corpus, their interdependence, and their importance according to the year. In the last section, a global graphic representation of the words is provided, showing their frequency and co-occurrence.

### 3.1. Frequency and word cloud

A frequency analysis was undertaken to provide an overview of the analyzed data: number of texts, number of total words, and grammar categories, etc. Some of these data are reflected in Table 1. The analyzed corpus was made up of 11 texts, corresponding to the 10 years analyzed. In total, 204.232 words were obtained (number of occurrences), of which only 4.561 appeared once (hapax number). The number of forms (number of verbs, adjectives, nouns, etc.) was 15.918. When reduced to its generic representation or root (lemmatization), it was 10.108. With these data, the lexical wealth was calculated, dividing the number of forms by the number of word occurrences and multiplying them by one hundred to find the percentage (Capsada Blanch and Torruella Casañas, 2017). A result of less than 20% means the corpus is extensive enough for analysis (Bolasco, 1999); in our case, it was 4.95%.

**Table 1:** Analysis of the text corpus about FPV.

Number of texts (selected from newspapers)	11
Number of classified text segments (93%)	5.337
Total number of text segments	5.739
Number of word occurrences (N)	204.232
Number of forms	15.918
Different words (V)	10.108
Hapax V1	4.561
Lexical wealth (V/N)* 100	4.95%
Linguistic refinement (V1/V)* 100 (forms)	45.12%
Linguistic refinement (V1/V)* 100 (words)	2.23%
Average occurrence per text	17.01933

The symbol * 100 means the percentage value.

**Figure 1:**
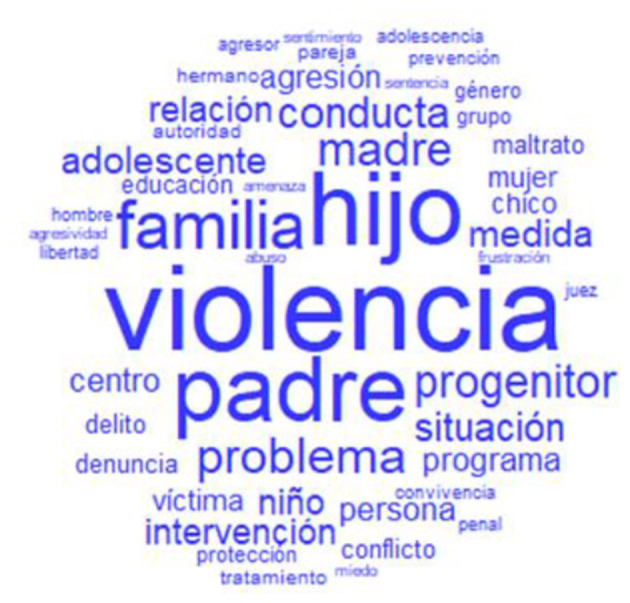
Word cloud from the corpus about FPV.

Some of the words that appeared most frequently were “violence” (1,410 times), “son” (1,213 times), “father” (1,057 times), “family” (749 times), “case” (657 times), “problem” (503 times), “mother” (480 times), “parent” (437 times), “adolescent” (347 times), and “child” (318 times), among others. These words can be seen in the “word cloud” depicted in Figure 1. The “word cloud” groups graphically organize the most frequent words, which appear in the center and in a larger size; the rest disperse and decrease in size as their frequency decreases. This figure allows the quick identification of keywords in a given corpus.

### 3.2. Factorial analysis of correspondence (IRaMuTeQ)

**Figure 2:**
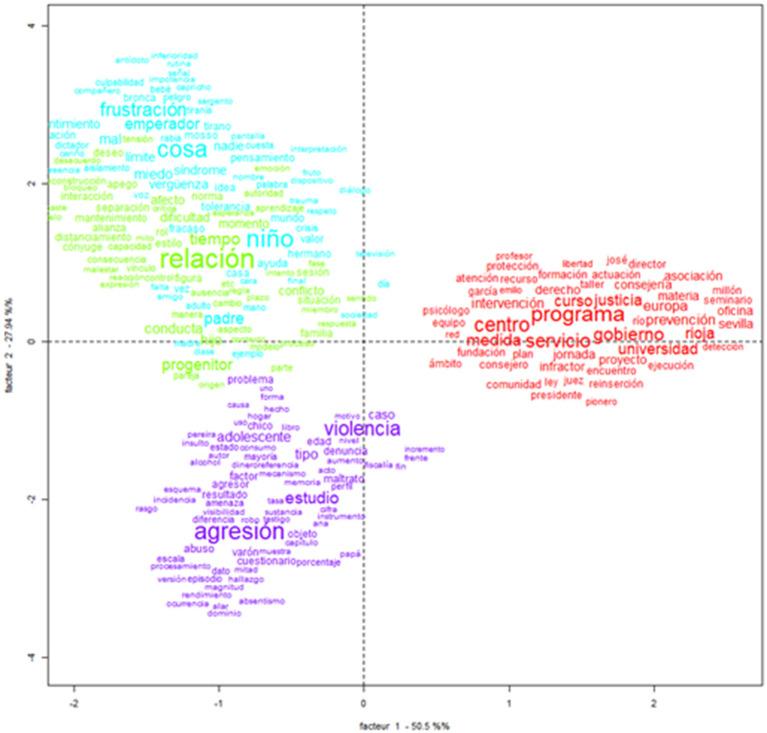
Factorial analysis of the lexical correspondence of the corpus on FPV.

The factorial analysis of correspondence allowed the semantic contextualization of the object of study, as well as the visualization of the distances between the textual corpus. As shown in Figure 2, two factors, which are represented on the X and Y axes, were observed: the first factor explains 50.5% of the variability of the total text corpus, while the second factor shows 27.94%. The two-dimensional view clearly presents four distinct areas, which are directly associated with the four classes previously presented.

### 3.3. Descending hierarchical classification (Reinert method)

**Figure 3:**
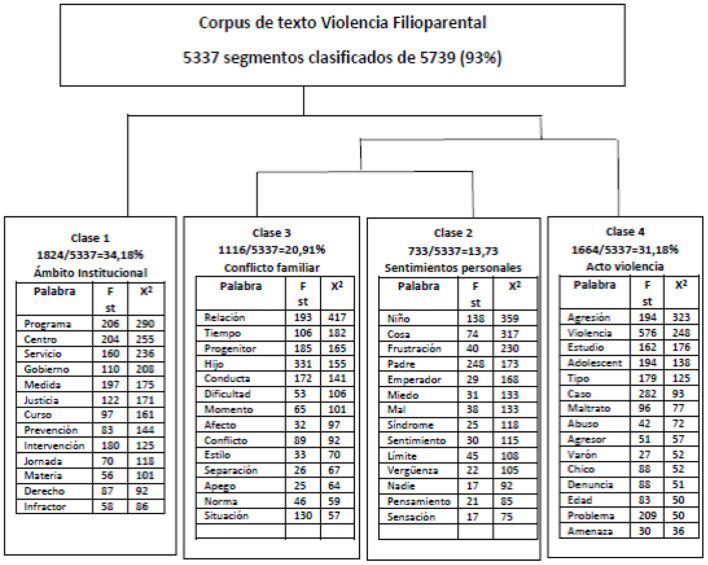
Descending hierarchical classification (Dendrogram Reinert method).

The results obtained, both in the descending hierarchical classification and those described in the factor analysis of lexical correspondence, could be considered the most interesting of the study. The descending hierarchical classification, as shown in Figure 3, defined lexicon classes that were represented by a subject; each class of the four that appeared could be defined based on the scope it dealt with. It was interpreted as shown in Figure 3. In this case, the first class was named “institutional environment”—it was composed of 1824 text segments units out of the total of 5,337, which makes a 34.2% explanation of the variability of the corpus. The words focused on the institutional part: “programs,” “centers,” “justice,” etc. The third class was named “familiar conflict,” with a total of 1116 text segment units, which becomes a 20.9% explanation of the variability of the corpus. We circumscribed terms to everything related to the relationships between children and parents and their consequences, such as “parent,” “son,” “relationship,” “time,” “behavior,” or “conflict.” The second class was named “personal feelings,” with a total of 733 text segments, which makes a 13.7% explanation of the variability of the corpus. In this case, we focused on the purely individual sphere, with terms such as “child,” “father,” “tyrant,” and “frustration”. Finally, a fourth class named “violence act” appeared with 1,664 text segment units, representing 31.18% of the variance. It describes the process of “violence” with terms such as “aggression,” “mistreatment,” and “abuse”.

### 3.4. Similarity analysis of the text corpus

**Figure 4:**
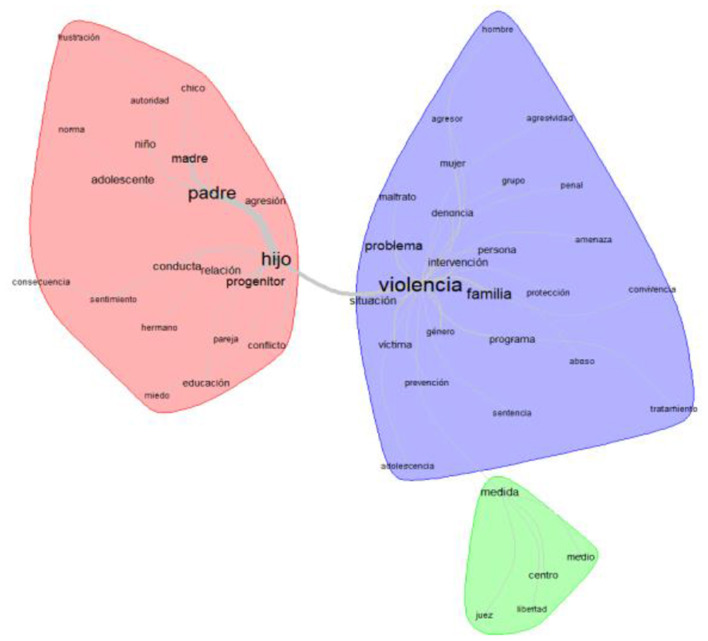
Similarity analysis of the text corpus about FPV.

In order to analyze the co-occurrence between terms, we used the similarity analysis technique. This analysis makes use of a graph that represents the connection between words in the analyzed textual corpus, which makes it possible to identify the existing co-occurrences between them. It also helps to identify the structure of a textual corpus and distinguishes common parts and specificities. As shown in Figure 4, in our study, we observed three blocks of words or groups of related words: on the one hand, the concept of “father” is related to “son,” “mother,” “child,” “boy,” and “adolescent,” and on the other hand, the concept of “violence” is related to “problem,” “family,” “aggressor,” “mistreatment,” “victim,” “sentence,” and “person.” We can deduce that in this “cluster” the words are related to the child's violence toward their parents. Finally, there is a category that refers to the consequences of abusive behaviors toward parents: a third, smaller group referring to judicial issues such as “center,” “measure,” “judge,” and “middle.”

## 4. Discussion

The results obtained in this study show that the press in general, and the Spanish press more specifically, reflect in their texts the contemporary reality. In the case that concerns us, FPV is captured in a different way in 2010 than in 2020. The main objective of this study was to analyze the vocabulary used in the different newspapers that refer to FPV and the relationship and concordance between the words that comprise it. In the analysis carried out, the lexicon takes on a special relevance, since it expresses contents or concepts by which the meaning of words is studied, as well as the different relationships that are established between the terms. In this work, FPV appears to be associated with terms such as father, mother, child, interpersonal relationships, authority, government, and measures, among others.

The examined newspapers differ in their ways of approaching this phenomenon, since they do so from different spheres, such as the family, judicial, institutional, and academic spheres. Likewise, in the division of the “clusters,” similarities were observed between them, since terms such as “violence,” “son,” and “father” are among the terms most frequently most used by all journalists.

The frequency analysis provided a quick overview of the most frequently used words over the last decade. The most frequently repeated word is “violence,” after which follow others such as “father,” “son,” or “family”, which are the most frequently occurring after “violence” but nonetheless remain far behind in the number of times they are repeated. When looking at these results, one might note that while the word “father” appears as the second most used word, the word “mother” is the sixteenth.

With the analysis of specificities, we observed the variations in frequencies according to the year. The most striking change in this analysis was the great presence of the concept of “violence” in a dominant way throughout much of the decade. However, in 2020 the trend changed, and the most used word became “father.” In the factorial analysis of correspondences, the two factors were presented, and the distance between the corpora was observed. It is clearly seen how the four clusters remain independent: on the one hand the institutional section, on the other the one related to people and their processes, and thirdly the one related to the facts, violence, complaint, and abuse.

The descending hierarchical classification shows these “clusters” or lexical classes: the first class focused on the institutional–judicial sphere, with terms such as “program,” “government,” “center,” or “justice”. The second class focused on everything related to personal feelings, with terms such as “frustration,” “fear”, “shame”, “syndrome,” or “bad” centered on the “child” or the “father”. The third class was related to family conflict, with terms such as “relationship,” 'parent,” “child,” “behavior,” “difficulty,” “attachment,” or “separation”. Finally, the fourth class focused on the act of “violence,” with elements such as “aggression,” “mistreatment,” “abuse,” “aggressor,” “denunciation,” or “threat.”

This classification reflects the characteristics of the phenomenon, which has only recently been investigated in the literature.

With regard to the first cluster, and in particular the judicial effects of the phenomenon, the mass media tend to confuse the judicial and institutional consequences of the phenomenon of child-to-parent aggression with those of adolescent-to-parent aggression. However, as in early childhood aggression, less harm is caused, and the consequences are not as serious. The parental role in this developmental stage is different from that of young people, as are the legal consequences for children and parents. Furthermore, aggression by young children hardly results in physical injury, although it may cause emotional distress to parents and continues into adolescence and adulthood, the latter in the sense of violence against an intimate partner (Ibabe, 2020).

In relation to personal feelings—the second cluster—internal factors such as shame and external factors such as social judgment on parenting skills lead the victim to deny or minimize the phenomenon (Sicurella, 2018). Furthermore, Cottrell and Monk (2004) argue that the reluctance to disclose these problems is exacerbated by limited access to means of intervention by local services. The sense of isolation, stigma, and shame that parents feel is, in fact, exacerbated by the lack of recognition and suitable policies, as also highlighted by the articles examined, as well as by the lack of awareness—in some cases—of the problem.

In relation to family conflict—the third cluster—the literature has highlighted how exposure to physical and verbal punishment by parents could influence the aggressiveness of children in the family context. For example, longitudinal studies on adolescents underline that the risk of CPV is related to prior parental aggression and, specifically, that mother-to-child physical aggression is the strongest indicator of physical CPV. Furthermore, adolescents in families with parental violence have been shown to manifest more levels of CPV when compared to other families (Ibabe et al., 2013; Contreras and del Carmen Cano, 2016). In other words, the results support the idea that child-to-parent violence is a consequence of parent-to-child violence. What remains to be determined is if reciprocal effects (simultaneous or close in time) may also explain the relationship between child-to-parent and parent-to-child violence (Gallego et al., 2019).

The last cluster refers to the multiple definitions given by the mass media and the scientific community to the term “violence” (Gallego et al., 2019). These definitions include those restricted to physically violent behavior or threats (Agnew and Huguley, 1989) and to others, including psychological violence (Calvete et al., 2015); other definitions require intent to cause injury (with the exclusion of pathologies, illegal substance abuse, homicide, or attempted homicide, without a previous history of violence), but other definitions do not (Loinaz et al., 2017).

Finally, the analysis of similarities in the present research underlines the frequency of the words and the frequency with which they appear related to one another. As expected, the most frequent words are those related to people, father, and son, which are the most interrelated. That is, on one hand, the concept of “father” is related to “mother,” “son,” “boy,” or “adolescent.” On the other hand, the concept of “violence” is related to “problem,” “behavior,” “victim,” “aggression,” “complaint,” “aggressor,” “harassment,” or “complaint,” among others.

## 5. Conclusion

The prevalence of this phenomenon, as mentioned above, follows an upward trend. There has been an increase in cases of violence between children and parents, which is reflected in the number of complaints by parents who claim to have been attacked and abused by their children. However, more conclusive studies are needed on this type of violence in Spain (Aroca et al., 2014), and the lack of them makes it more difficult to approach this intrafamily process.

Several qualitative studies have been conducted in the United States, Canada, and Australia. However, in Spain, if we exclude the most striking episodes—such as the cases of homicide or attempted murder, which are disclosed by the mass media—there is a lack of significant research experience on this specific topic capable of providing detailed information with respect to this increasingly widespread phenomenon; this is also due to changes in the family structure, educational styles, and rhythms of life. The mistreatment perpetrated by children in the family is, therefore, still a submerged problem that has not been tackled with scientific accuracy.

Western society is changing daily and so are the social structures, mainly that of the family. The nuclear family as the predominant model has given way to other models, including families where parents are of the same gender and the single-member family, headed by either a father or mother. These models must be considered when explaining such behaviors and their relationship with the actions performed by any member of the family. It must also be kept in mind that most of the studies reviewed focus on a single family member, and different family members produce different narratives of domestic violence situations.

Furthermore, there are authors who, in examining the causes of the phenomenon of violence against parents, identify the existence of personality disorders (such as low self-control, low tolerance for frustration, lack of empathy, low self-esteem, egocentricity, hyperactivity), in doing so dismantling the causality between mental health and FPV (Ibabe et al., 2007; Rechea and Cuervo, 2009; Ibabe and Jaureguizar, 2011), although the role of the family context remains the main predictive factor.

However, it should not be forgotten that the information disseminated by the media may be biased by opinions, editorials, and political ideologies. In this study, we searched for news about FPV and did not calculate the balance between one newspaper and another. There are newspapers that dedicate more lines to this problem, while others only more briefly mention the cases that have occurred. The different ideological currents were also not considered in the analysis of the information, since what was sought was to extract information and see how relevant FPV is in the Spanish press.

Though this study was intended to be exhaustive, it has some limitations. One is the fact that the sample includes mostly national newspapers and three regional newspapers that are distributed only in the Canary Islands, such as “Canarias7,” “La Provincia,” and “El Día.” This has created a bias in the sample in two ways. Firstly, if we bear in mind that a national newspaper is going to present a greater number of articles than a regional one, a national newspaper will thus have a greater weight in the reporting of the events, meaning that the regional paper will influence the results to a lesser degree. Secondly, the way newspapers from autonomous communities other than the Canary Islands disseminate news related to FPV cannot be discerned. For future research, complementary studies could be carried out to analyze the differences between national and regional newspapers. By doing so, it could be clarified whether the differences in written coverage vary more depending on the years or on the newspaper, using both the year variable and the periodic variable for the analysis.

In addition, by taking advantage of the information collected in the reports of the State Attorney General's Office on the prevalence of FPV in the autonomous communities, a comparative study of the communities with the largest and smallest number of files and the information presented to the media could be conducted. Likewise, it would be of great interest to the general population to fill out a questionnaire on the subject, in order to find out to what extent people endorse the social and cultural norms related to FPV that are transmitted not only the media, but also on social media, radio, or television programs.

Despite the above limitations, this study contributes to the current knowledge of FPV. It demonstrates that the extent of this phenomenon can be influenced by the source of information, therefore underlining the importance of integrating different sources. In general, a trend has been observed to minimize problems between parents, which can negatively affect the attempts to fix them.

Even so, the results of this study could be of considerable importance from a psychological and prevention perspective. Intervention efforts to reduce rates of violence should focus on helping parents to manage conflictive relationships with their children, or at least on educating them about the importance of buffering children from exposure to conflict and on presenting strategies for improving family cohesion and organization.

Regarding the age variable, there is a diversity of results, although most studies place the onset of FPV in adolescence (Martinez, 2015) and have established different intervals, depending on the source: from 9 to 13 years (social services), from 10 to 15 years (investigations), and from 14 to 17 years (prosecutor for minors). As aforementioned, research carried out through the analysis of complaints of violence indicates that the peak of FPV is when the children are between 14 and 17 years old, frequently decreasing at older ages (Simmons et al., 2018).

The literature confirms that patterns of violence and victimization can develop in early adolescence, quickly becoming difficult to correct. Consequently, primary prevention measures play an essential role in the fight against violent behavior manifested by children toward parents; in particular, prevention in adolescence and preadolescence is more effective if carried out through schools, the mass media, and newspapers, as they are a fundamental component of adolescent life and the main contexts in which gender socialization takes place, as well as places where behaviors toward oneself and others are formed and strengthened.

In all likelihood, the increase in this phenomenon in adolescence is linked to the acquisition of self- identity, which is considered the main developmental task of adolescence, in which individuals struggle to assert themselves as an entity while seeking to maintain a bond with their past and family context and accepting the values shared by a group (Pellerone et al., 2017a,b).

Furthermore, future research should also consider CPV in young adults (subjects above the age of 18), because many young people still live with their parents after becoming legal adults, although it is not necessary for parents and children to reside together for abuse to happen.

Since risk factors and patterns of abuse may evolve with age, it is important to study different development phases, starting with children under 14 years of age—whose cases are seldom processed by the mass media—up to young adulthood.

In conclusion, in samples of Spanish teenagers, young people, and adults, it is common to find more psychological violence perpetrated by girls than boys, with no gender difference detected in physical violence. However, other studies have found higher rates of parental violence in young boys, leading to the hypothesis that CPV patterns change with the age of the young person.

## Data availability statement

The original contributions presented in the study are included in the article/supplementary material, further inquiries can be directed to the corresponding author.

## Author contributions

JT, MW, and MP have made substantial, direct, and intellectual contributions to the work. All authors have approved it for publication.
